# WD repeat domain 6 as a novelty prognostic biomarker correlates with immune infiltration in lung cancer: A preliminary study

**DOI:** 10.1002/iid3.681

**Published:** 2022-08-17

**Authors:** Minghe Lv

**Affiliations:** ^1^ Department of Radiation Oncology, Fudan University Shanghai Cancer Center Fudan University Shanghai China; ^2^ Department of Oncology, Shanghai Medical College Fudan University Shanghai China

**Keywords:** biomarker, immune infiltration, lung cancer, WDR6

## Abstract

**Background:**

WD repeat domain 6 (WDR6), a novel human WD‐repeat gene, encodes a member of the WD repeat protein family, and its tumorigenic effect has rarely been reported so far.

**Methods:**

Our study used Oncomine, TIMER2.0, GEPIA2, Kaplan–Meier plotter, PrognoScan, and TISIDB tools to analyze the differential expression between pan‐cancer, especially lung cancer, and corresponding normal tissue, and further explore the prognostic and immunological role of WDR6 expression.

**Results:**

Our results showed WDR6 was lower expressed in lung squamous cell carcinoma than in normal tissue, but WDR6 expression was correlated obviously with clinical stage in Lung adenocarcinoma. The overall survival, first progression, postprogression survival, and Relapse‐free survival of lung cancer patients were longer in the WDR6 high‐expression group than in the low‐expression group. We found the expression of WDR6 significantly correlated with immune molecules, including immunomodulators, lymphocytes, and chemokines in lung cancer.

**Conclusion:**

WDR6 can be used as a prognostic marker for lung cancer and is significantly associated with immune cell infiltration.

## Graphical abstract

In this study, we first explored the prognostic and immunological role of WD repeat domain 6 (WDR6) on lung cancer based on the public datasets. The results concluded that there is a possible novel prognostic molecular marker for good survival correlated with immune cell infiltration in lung cancer, called WDR6 expression. This study first offers a relatively comprehensive understanding of the oncogenic roles of WDR6 for lung cancer.

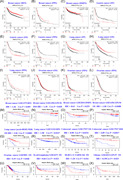

## 1 INTRODUCTION

Lung cancer is one of the most common cancers worldwide and a leading cause of cancer‐related death. Its morbidity and mortality rate ranks first among all the major tumors.^1^ Non‐small cell lung cancer (NSCLC) accounts for about 80‐85% of newly diagnosed lung cancer cases, and in NSCLC, Lung adenocarcinoma (LUAD) and lung squamous cell carcinoma (LUSC) are the two most common pathologic types. Although the current treatment methods for lung cancer are changing rapidly, the 5‐year survival rate for patients with NSCLC is less than 15%−20%. Therefore, seeking new biomarkers for early detection and diagnosis of lung cancer is very important for the treatment of lung cancer patients.

WD‐repeat protein is a conserved core of about 40 amino acids consisting of 4 or more repeated units, usually ending with tryptophan‐aspartic acid (WD), belonging to a large and rapidly expanding conserved protein family.^2^ The consequence of these proteins is not only reflected in their key roles in signal transduction, transcriptional regulation,^3^ apoptosis, and many other important biological functions but also, in their association with a number of human diseases, such as late‐onset sensorineural deafness phenotype and triple‐A syndrome.^4^ WD repeat domain 6 (WDR6), a novel human WD‐repeat gene, encodes a member of the WD repeat protein family, and this protein includes in 1121 amino acids and contains 11 WD‐repeat units. Many aspects of the function and role of WDR6 have not been described in detail, especially the role of WDR6 in tumors. Therefore, we aimed to explore the effects of WDR6 expression on pan‐cancer by employing public datasets, such as the cancer genome atlas (TCGA), Gene expression omnibus, PrognoScan, TISIDB, and LinkedOmics, which contain functional genomics databases for different cancers, mainly to investigate its prognostic and immunological role for LUAD and LUSC in this study.

## 2 METHODS AND MATERIALS

### 2.1 Gene expression analysis

We used the TIMER2.0 (Tumor immune estimation resource, version 2) and GEPIA2 (Gene Expression Profiling Interactive Analysis, version 2) tools to explore the WDR6 expression difference between pan‐cancer and normal tissues and the correlation between WDR6 expression and the pathological stages (stage Ⅰ, stage Ⅱ, stage Ⅲ, and stage Ⅳ) of cancers.

### 2.2 Survival prognosis analysis

We employed Prognoscanand Kaplan−Meier plotter databases to study the effects of WDR6 expression on prognosis in different cancers. We also used the Kaplan−Meier plotter tool to investigate the impact of both clinicopathological factors and WDR6 on patient outcomes in lung cancer patients.

### 2.3 Immune infiltrating analysis and prognosis analysis

We used the TIMER web server to gain the relationship between the expression of WDR6 and the infiltration of CD8^+^ T cell, CD4^+^ T cell, macrophage, neutrophil, and dendritic cell in LUAD and LUSC. The *p* values and partial correlation (cor) values were obtained via the purity‐adjusted Spearman's rank correlation test. The data were displayed by a scatter plot. The correlation between the gene markers of immune infiltrating cells and WDR6 expression was analyzed by GEPIA2 and TIMER2.0 tools.

### 2.4 TISIDB database analysis

TISIDB Database is a web for cancer and immune system interactions that integrates multiple heterogeneous data types. The TISIDB data set was used to analyze the relationship between WDR6 expression and lymphocytes, immune modulators (including immunosuppressants and immunostimulants), and chemokines.

### 2.5 Statistical analysis

The data from the Oncomine database were presented as *p* values determined in *t*‐tests, fold changes, and gene ranks. In their respective analyses, survival maps were generated using the PrognoScan, Kaplan−Meier Plotter, TIMER, TIMER2.0, and GEPIA2 databases, including HR and *p* values or *p* values from log‐rank tests. Spearman's and Pearson's correlation analyses were used to measuring the degree of correlation between specific variables. *p* < .05 was considered statistically significant if not especially noted.

## 3 RESULTS

### 3.1 The differential expression of WDR6 gene between pan‐cancer and normal tissue

**Figure 1:**
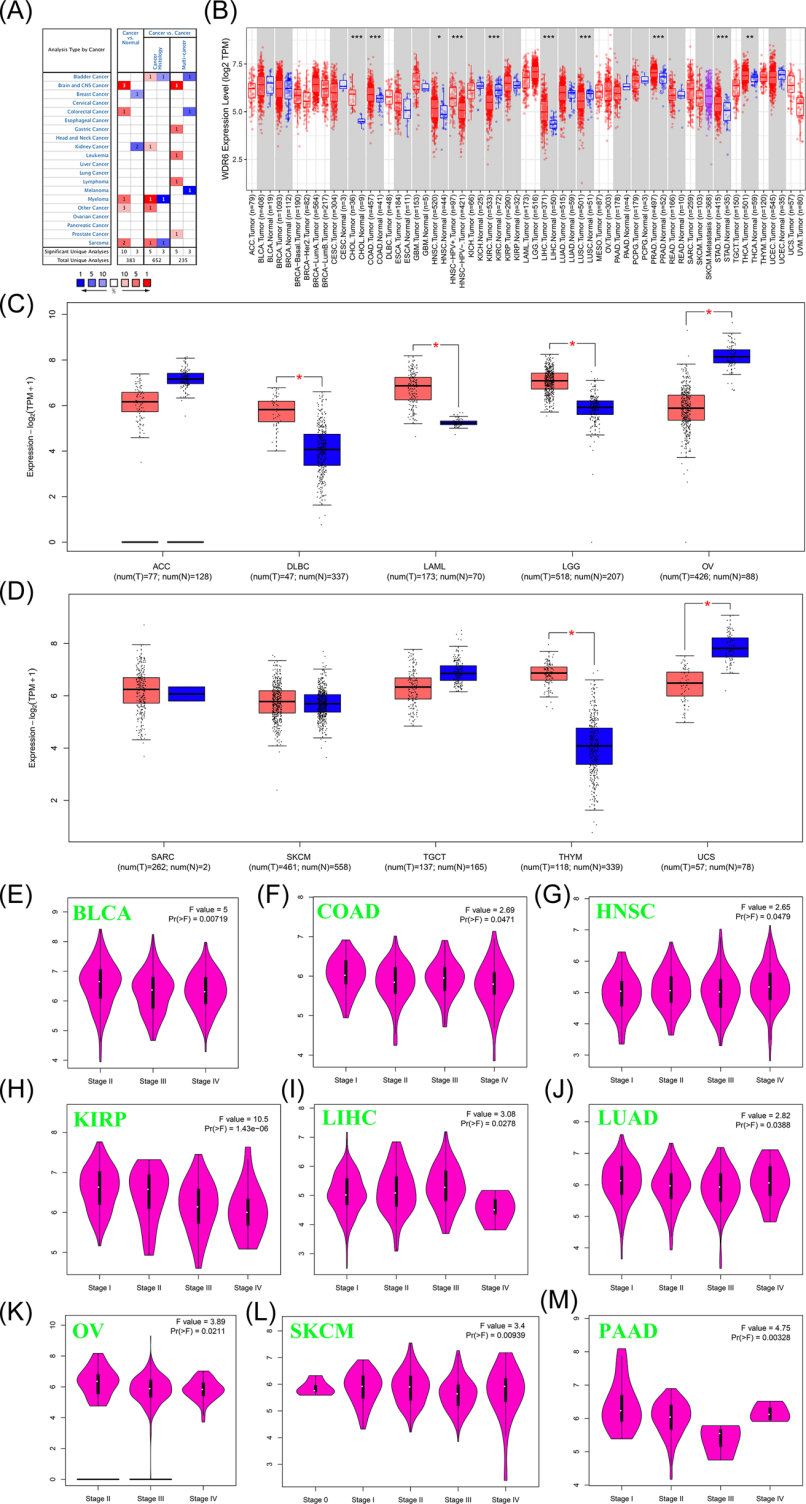
The expression of the WDR6 gene between pan‐cancer and normal tissues in TCGA and Oncomine datasets. We used the data from Oncomine (A) and TIMER2.0 (B) datasets to analyze the expression difference of WDR6 between pan‐cancer and normal tissue. We further supplemented the expression profile of WDR6 between ten other tumors and corresponding normal tissues by using the GEPIA2 tool (C and D). (E−M) The expression of WDR6 were analyzed by the main pathological stages in different tumors. *p < .05; **p < .01; ***p < .001. COAD, Colon adenocarcinoma; HNSC, Head and Neck squamous cell carcinoma; KIRC, Kidney renal clear cell carcinoma; LIHC, Liver hepatocellular carcinoma; LUAD, Lung adenocarcinoma; OV, Ovarian serous cystadenocarcinoma; SKCM, skin Cutaneous Melanoma; TCGA, the cancer genome atlas; WDR6, WD repeat domain 6.

To explore the expression pattern of WDR6, in the present study, we first analyzed the data of the Oncomine database, and the results showed that compared with corresponding normal tissues, the expression of WDR6 was decreased in breast and kidney cancer, but was increased in brain and CNS cancer, colorectal cancer, myeloma, and sarcoma (Figure 1A). We further analyzed the data from the TCGA data set by TIMER2.0 web, and the results showed that the expression of WDR6 was markedly increased in Cholangio carcinoma (CHOL), Colon adenocarcinoma (COAD), Head and Neck squamous cell carcinoma (HNSC), Liver hepatocellular carcinoma (LIHC), Prostate adenocarcinoma (PRAD), Stomach adenocarcinoma (STAD), and Thyroid carcinoma (THCA), compared to normal tissues, but was obviously decreased in LUSC and Kidney renal clear cell carcinoma (KIRC) (Figure 1B). Specially, we further used the GEPIA2 tool via matching TCGA normal and GTEx data to analyze the differential expression of WDR6 in Adrenocortical carcinoma, Lymphoid Neoplasm Diffuse Large B‐cell Lymphoma (DLBC), Acute Myeloid Leukemia (LAML), Brain Lower Grade Glioma (LGG), Ovarian serous cystadenocarcinoma (OV), Sarcoma (SARC), Skin Cutaneous Melanoma (SKCM), Testicular Germ Cell Tumors (TGCG), Thymoma (THYM), and Uterine Carcinosarcoma. As shown in Figure 1C−D, there was a lower expression of WDR6 in OV and UVM than in corresponding control tissues, but a higher expression of WDR6 in DLBC, LAML, LGG, and THYM. In addition, the correlation between WDR6 expression and the clinical stage was analyzed by using the GEPIA 2.0 tool, and the results showed that the expression of WDR6 in BLCA, COAD, HNSC, KIRP, LIHC, LUAD, OV, SKCM, and PAAD correlated obviously with clinical stage, respectively (Figure 1E−M).

### 3.2 Prognosis analysis of WDR6 expression in Kaplan−Meier plotter and PrognoScan databases

**Figure 2:**
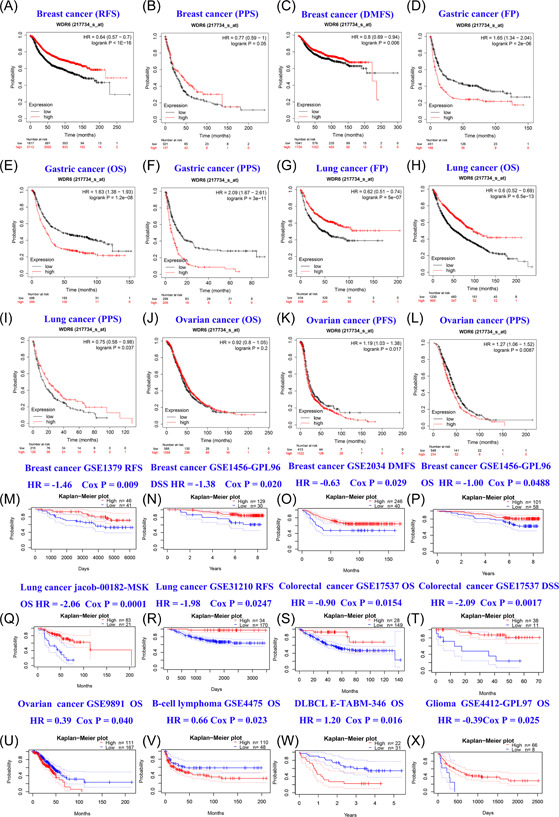
The prognosis analysis of WDR6 expression in Kaplan−Meier Plotter and PrognoScan databases. (A−K) The Kaplan−Meier curve showed the results of survival analysis in breast, gastric, lung, and ovarian cancer by using the Kaplan−Meier plotter tool. (L−W) The survival data from PrognoScan data set was analyzed by the Kaplan−Meier curve. DLBCL, Diffuse Large B Cell Lymphoma; DMFS, distant metastases‐free survival; DRFS, Distant Recurrence Free Survival; DSS, Disease Specific Survival; FP, first progression; OS, Overall Survival; PFS, Progression‐free Survival; PPS, postprogressison survival; RFS, Relapse‐free survival.

To further study the effect of WDR6 on prognosis in pan‐cancer, we also analyzed the survival data from Kaplan−Meier plotter and PrognoScan databases between the WDR6 high‐expression group and WDR6 low‐expression group. The results showed that in Kaplan−Meier plotter and PrognoScan databases, Relapse‐free survival (RFS), postprogression survival (PPS), and distant metastases‐free survival (DMFS) of breast cancer patients in the WDR6 high‐expression group were all significantly longer than the WDR6 low‐expression group (Figure 2A−C). However, as shown in Figure 2D−F, we found that in gastric cancer, there were shorter the first progression (FP), OS, and PPS in WDR6 high‐expression group than in the WDR6 low‐expression group. The FP, OS, and PPS in the WDR6 high‐expression group for lung cancer patients were obviously longer than in the WDR6 low‐expression group (Figure 2G−I). As for ovarian cancer patients, the results exhibited that the overall survival (OS) of the WDR6 high‐expression group was no more significant than the WDR6 low‐expression group (Figure 2J); however, there were significantly longer PFS and PPS in the WDR6 low‐expression group than in the high‐expression group (Figure 2K−L). All survival analysis data from the Kaplan−Meier plotter data set of this part manifested that WDR6 could be a potential and poor prognostic factor for gastric cancer and ovarian cancer patients, but a better prognostic biomarker for breast cancer and lung cancer patients. To further verify the effect of WDR6 gene expression on prognosis, we employed the data from the PrognoScan database to investigate whether WDR6 expression could lead to a better prognosis for special types of cancers. As shown in Figure 2M−P, The RFS, DSS, DMFS, and OS of breast cancer patients in the WDR6 high‐expression group were significantly longer than the WDR6 low‐expression group, which kept in with the survival data from the Kaplan−Meier plotter data set, and these data further suggested that WDR6 served as a potential and favorable biomarker on clinical diagnosis and therapy for breast cancer. Similarly, as for lung cancer, the result of OS in the PrognoScan database sustained the conclusion that in lung cancer patients, there was markedly longer OS in the WDR6 high‐expression expression group than in the WDR6 low‐expression group, suggesting that WDR6 could be a great prognostic biomarker for lung cancer (Figure 2Q). As shown in Figure 2R, we also found that the DSS of lung cancer patients in the WDR6 high‐expression group were lengthened compared to the low‐expression group, which WDR6 could served as a specific factor for disease‐specific survival. Furthermore, we found the same effect on the prognosis and survival in colorectal cancer, and the OS and DSS of the WDR6 high‐expression group were longer than the low‐expression group (Figure 2S‐T). However, in the ovarian cancer survival data from the PrognoScan data set (*n* = 1656), we found that the OS in WDR6 high‐expression group was not significant as in the low‐expression group, which contradicted the analysis of survival data from the Kaplan−Meier plotter data set (*n* = 278) (Figure 2U), and we speculated that the contradictory results could be caused by the number of samples, and the effect of WDR6 expression on the OS of ovarian cancer still needed further investigation in detail. As shown in Figure 2V−W, we found that the OS of blood cancer patients including B‐Cell Lymphoma and DLBCL in the WDR6 high‐expression group were significantly shorter than the low‐expression group. The prognostic difference between the WDR6 high‐expression group and the low‐expression group of glioma patients was displayed in Figure 2X, which indicated that WDR6 expression could be of benefit to great prognosis for glioma. To investigate the effect of WDR6 expression on survival prognosis, we also divided the data from TCGA into high‐expression and low‐expression groups via employing the GEPIA 2 tool, the results also showed that the expression of WDR6 would make a significant effect on OS and disease‐free survival (Supporting Information: Figures S1A and 1B).

### 3.3 The effect of different clinicopathological factors on the expression of the WDR6 gene and clinical prognosis in lung cancer

As shown in Table 1, we found that High WDR6 expression predicted better OS and FP in both male and female lung cancer patients. As for early‐stage lung cancer, such as T1, T2, N0, or N1, the role of WDR6 expression on survival prognosis showed a more significant effect. Compared with LUSC patients, LUAD patients with high WDR6 expression showed a greater overall survival. High WDR6 expression in both smokers and nonsmokers suggested a better prognosis. The data of this part indicated that WDR6 could be a novel and prognostic biomarker for tumor diagnosis and therapy in lung cancer patients, especially for early LUAD.

**Table 1:** Kaplan−Meier plotter to determine the effect of different clinicopathological factors on the expression of the WDR6 gene and clinical prognosis in lung cancer

Clinicopathological characteristics	Overall survival (*n* = 1925)			First progression (*n* = 982)		
	*N*	Hazard ratio	*p* Value	*N*	Hazard ratio	*p* Value
Sex						
Male	1100	0.63 (0.52−0.76)	****	314	0.68 (0.52−0.88)	**
Female	714	0.55 (0.4−0.71)	****	468	0.54 (0.4−0.72)	****
Stage						
I	577	0.39 (0.29−0.52)	****	325	0.33 (0.2−0.54)	****
II	244	0.66 (0.46−0.94)	*	130	1.6 (0.84−3.01)	0.15
III	70	1.41 (0.78−2.54)	0.25	19	−	−
IV	4	−	−	0	−	−
Stage T						
1	437	0.51 (0.36−0.71)	****	177	0.58 (0.3−1.11)	0.094
2	589	0.74 (0.59−0.93)	**	351	0.86 (0.64−1.17)	0.34
3	81	1.53 (0.87−2.68)	0.14	21	0.14 (0.03−0.67)	**
4	46	0.57 (0.29−1.11)	0.094	7	−	−
Stage N						
0	781	0.62 (0.49−0.77)	****	374	0.7 (0.51−0.98)	*
1	252	0.66 (0.47−0.93)	*	130	1.42 (0.88−2.29)	0.15
2	111	1.31 (0.87−1.98)	0.19	51	0.69 (0.34−1.37)	0.28
Stage M						
0	681	0.59 (0.46−0.77)	**	195	0.59 (0.36−0.98)	*
1	10	−	−	0	−	−
Histology						
Adenocarcinoma	719	0.45 (0.34−0.62)	****	461	0.41 (0.3−0.56)	****
Squamous cell carcinoma	524	0.89 (0.69−1.15)	0.37	141	0.46 (0.27−0.77)	***
Grade						
I	201	0.63 (0.43−0.92)	*	140	0.7 (0.43−1.12)	0.14
II	310	0.79 (0.58−1.09)	0.15	165	0.64 (0.37−1.1)	0.11
III	77	0.69 (0.35−1.35)	0.27	51	1.81 (0.61−5.33)	0.28
Smoking history						
Yes	820	0.68 (0.55−0.84)	****	603	0.74 (0.58−0.94)	*
No	No	0.52 (0.3−0.91)	*	193	0.35 (0.2−0.64)	****

Abbreviation: WDR6, WD repeat domain 6;

* *p* < .05

** *p* < .01

*** *p* < .001

**** *p* < .0001.

### 3.4 The association between the expression of WDR6 and immune infiltration as well as cell markers

**Figure 3:**
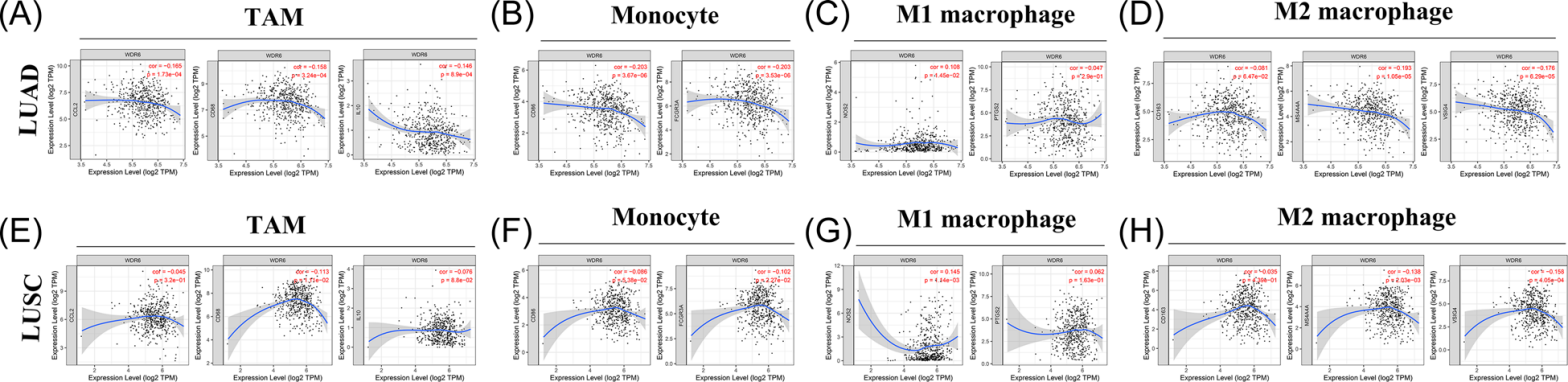
Association between WDR6 expression and immune cell markers in LUAD and LUSC. We used the TIMER tool to explore the correlation of WDR6 expression with TAM (A, E), monocyte (B, F), M1 macrophage (C, G), and M2 macrophage (D, H) markers in LUAD and LUSC. TAM, tumor‐associated macrophage; WDR6, WD repeat domain 6.

In this part, we further investigated whether WDR6 expression was associated with immune cell infiltration in lung cancer patients, finding that the expression of WDR6 obviously related to CD8^+^ T cell and CD4^+^ T cell in LUAD, while only correlated with CD4^+^ T cell in LUSC (Supporting Information: Figure S2). This study exhibited that in LUAD, the WDR6 expression negatively correlated with the tumor‐associated macrophages (TAM) markers, including CCL2, CD68, and IL10, and monocyte markers, such as CD86 and FCGR3A (CD16); however, there was no statistically significant correlation between the expression of WDR6 and M1 or M2 macrophages markers, such as NOS2 and CD163 (Figure 3A−D). As shown in Figure 3E−H, WE found that WDR6 expression did not negatively and significantly correlate with those immune cell markers, except CD68, FCGR3A (CD16), NOS2, MS4A4A, and VSIG4. Furthermore, we used the GEPIA2 tool to further analyze the correlation between WDR6 expression and immune cell markers, including B cell, CD8^+^ T cell, TAM, monocyte, M1 and M2 macrophages, and T cell exhaustion. The results of Table 2 showed that in LUAD rather than LUSC, the expression level of WDR6 significantly correlated with the TAM and monocyte markers, including CCL2, CD68, IL10, CD86, and FCGR3A, and the conclusions obtained from these results were consistent with those obtained from the previous analysis by using TIMER tool. We also found that CD8^+^ T cell markers, such as CD8A and CD8B, negatively and markedly correlated with the expression of WDR6 in LUAD patients, while the two did not have a significant association in LUSC patients. Therefore, we concluded that WDR6 expression is closely correlated with the markers of immune cell infiltration, especially TAM and monocyte, thereby providing a potential research direction for WDR6 to affect patient prognosis by immune infiltration.

**Table 2:** Correlation analysis between WDR6 and relate genes and markers of B cell, macrophages, TAM, monocyte, and T cell exhaustion in GEPIA2

Description	Gene markers	LUAD				Gene markers	LUSC			
		Cancer		Normal			Cancer		Normal	
		Cor	*p*	Cor	*p*		Cor	*p*	Cor	*p*
B cell	CD20 (KRT20)	−0.084	0.066	0.07	0.6	CD20 (KRT20)	0.036	0.43	−0.0046	0.97
	CD19	0.083	0.07	0.048	0.72	CD19	0.15	****	0.13	0.37
	CD38	−0.1	*	0.14	0.31	CD38	−0.053	0.25	0.058	0.69
CD8^+^ T cell	CD8A	−0.11	*	0.0059	0.96	CD8A	−0.039	0.39	−0.42	****
	CD8B	−0.12	*	0.012	0.93	CD8B	0.043	0.35	−0.5	****
M1 Macrophage	NOS2	−0.016	0.73	0.25	0.057	NOS2	0.074	0.1	0.24	0.091
	COX (PTGS2)	−0.015	0.75	0.045	0.73	COX (PTGS2)	0.0046	0.92	−0.085	0.56
M2 Macrophage	CD163	−0.081	0.075	−0.33	****	CD163	−0.025	0.58	−0.078	0.59
	MS4A4A	−0.15	****	−0.31	*	MS4A4A	−0.099	*	−0.074	0.61
	VSIG4	−0.15	****	−0.35	***	VSIG4	−0.074	0.1	0.017	0.91
TAM	CCL2	−0.14	****	−0.15	0.24	CCL2	0.0046	0.92	−0.13	0.38
	CD68	−0.13	****	−0.19	0.14	CD68	−0.12	****	−0.099	0.49
	IL10	−0.11	*	−0.27	*	IL10	−0.066	0.15	−0.1	0.48
Monocyte	CD86	−0.23	****	−0.32	*	CD86	−0.11	*	−0.23	0.11
	FCGR3A	−0.2	****	−0.042	0.75	FCGR3A	−0.095	*	−0.086	0.55
T cell exhaustion	PD1 (PDCD1)	−0.0063	0.89	−0.0013	0.99	PD1 (PDCD1)	0.13	****	−0.013	0.93
	PD‐L1 (CD274)	−0.042	0.36	−0.034	0.8	PD‐L1 (CD274)	−0.01	0.82	−0.061	0.67
	CTLA4	0.029	0.53	0.22	0.1	CTLA4	0.097	*	0.065	0.65

Abbreviations: Cor, R‐value of Spearman's correlation; LUAD, Lung adenocarcinoma; LUSC, lung squamous cell carcinoma; None, correlation without adjustment; TAM, tumor‐associated macrophage.

* *p* < .05;

** *p* < .01;

*** *p* < .001;

**** *p* < .0001.

### 3.5 The correlation analysis between WDR6 and immune molecules

**Figure 4:**
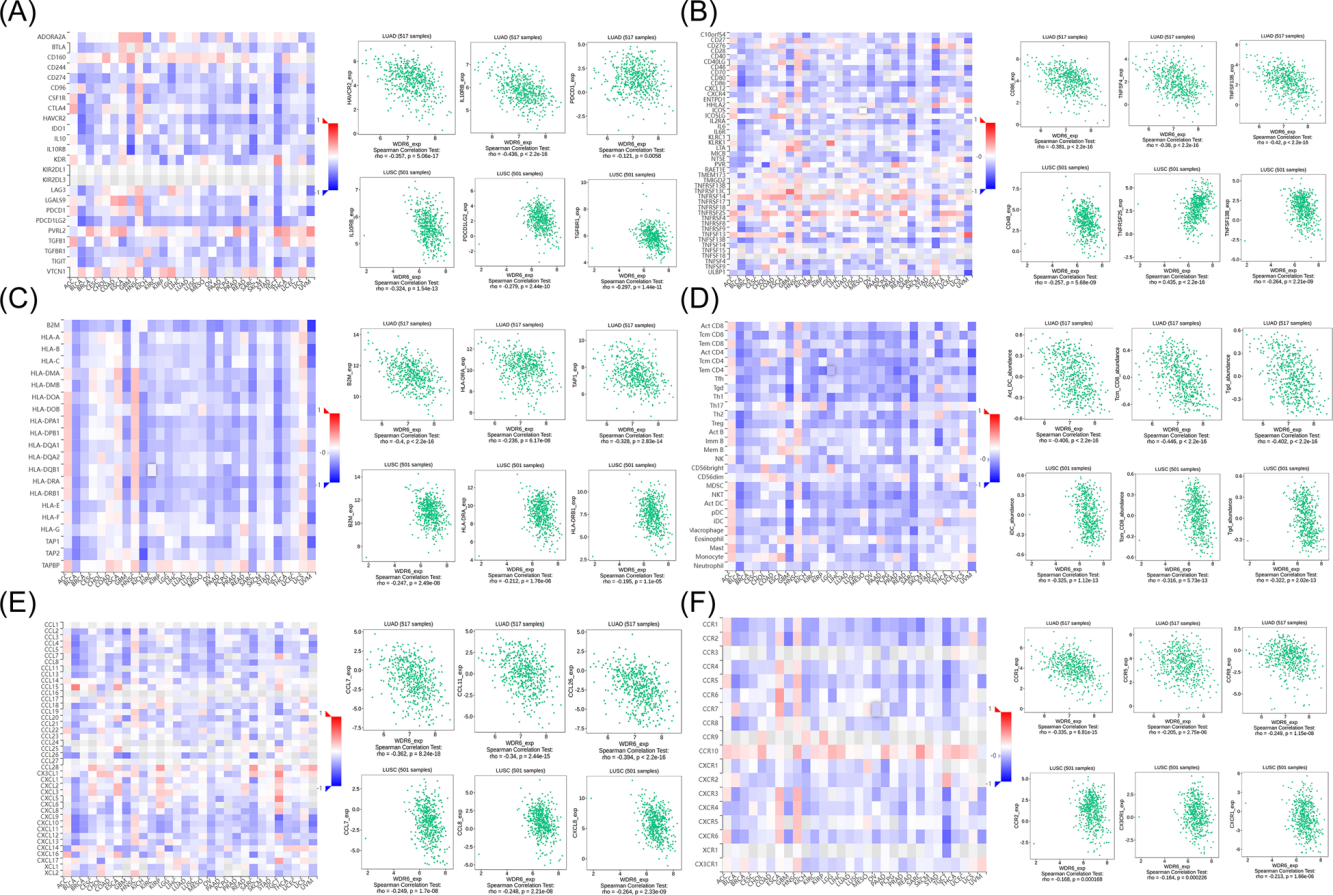
Correlation analysis of WDR6 expression with immunomodulators, lymphocytes, and chemokines in LUAD and LUSC from the TISIDB database. Relations of WDR6 expression with immunomodulators (A−C), TILs abundance (D) or chemokines (or receptors) (E−F) (plus the top 3 of highest correlation in LUAD or LUSC, respectively). LUAD, Lung adenocarcinoma; LUSC, lung squamous cell carcinoma; TILs, tumor‐infiltrating lymphocytes; WDR6, WD repeat domain 6.

In this part, we used the TISIDB dataset to further study correlations between WDR6 expression and abundant immune markers in LUAD and LUSC. In the TISIDB dataset, immunomodulators included immune inhibitors, immunostimulators, and major histocompatibility complex molecules. As shown in Figure 4A−C, THE heatmap respectively displayed the correlations between WDR6 and tumor‐infiltrating immune inhibitor, immunostimulator, and MCH molecules in pan‐cancer, and the scatter plots of the top 3 of the absolute value of *p* in LUAD patients, including PDCD1, IL10R, HAVCR2, CD86, TNFSF4, TNFSF13B, B2M, HLA‐DRA, and TAP1, and in LUSC patients including IL10RB, PDCD1LG2, TGFBR2, CD48, TNFRSF25, TNFSF13B, B2M, HLA‐DRA, and HLA‐DRB1 respectively were showed. The relationship of tumor‐infiltrating lymphocytes (TILs) in pan‐cancer was displayed in Figure 4D, AND the scatter plots of the top 3 of the absolute value of P in LUAD and LUSC patients showed that WDR6 expression negatively and obviously correlated with these TILs including Act DC, Tcm CD8, Tgd, and iDC. The relationship between WDR6 expression and chemokines in pan‐cancer was presented by heatmap, and especially, the top 3 scatter plots of the absolute *p* values showed the negative and significant correlation of the two in LUAD and LUSC (Figure 4E). Similarly, in Figure 4F, The association between WDR6 expression and receptors in pan‐cancer was also presented by heatmap, and the top 3 scatter plots of the absolute *p* values showed the correlation of the two in LUAD and LUSC. Therefore, it was confirmed that WDR6 participated widely in modulating various immune molecules in lung cancer patients especially LUAD to affect immune infiltration in the tumor microenvironment (TEM). In addition, we further investigated the co‐expression network of WDR6 in LUAD (Supporting Information: Figure S3) and LUSC (Supporting Information: Figure S4) from the LinkedOmics data set.

## 4 DISCUSSION

Up to now, the role of the WDR6 gene in tumors has hardly been reported. However, current studies showed that WD‐repeat proteins participated in signal transduction, transcription regulation, cell cycle regulation, and apoptosis, and were associated with human other diseases, such as Cockayne syndrome^3^ and Triple‐A syndrome. WD40 repeat domain proteins have been reporting to become a novel target class, and two proteins containing the WDR domain, WDR5, and EED, as well as other β ‐thruster domains, have been successfully targeted by potent, specific, cell‐active, drug‐like chemical probes.^5^ And studies have found that interaction with WDR5 which was a key determinant for MYC recruitment to chromatin, promoted target gene recognition and tumorigenesis by MYC,^6^ and WDR5 promoted proliferation and correlated with poor prognosis in esophageal squamous cell carcinoma,^7^ and overexpression of WDR5 associated with aggressive clinicopathological features and unfavorable prognosis in HNSC.^8^ Previous studies have shown that WDR6 was a newly discovered protein and may belong to a highly conserved subfamily of WD‐repeat proteins,^9^ and the expression of WDR6 was negatively associated with visceral specific metastasis in breast cancer patients (*r* = −0.319, *p* < .0001).^10^ In addition, WDR6 was found to be a possible target gene of miR‐451a in colorectal cancer and might play a crucial role in tumor regulation. However, there are no relevant studies on the effect of WDR6 expression on the prognosis of cancer patients, especially lung cancer. In this study, we elaborated for the first time that WDR6, a novel gene of the WD‐repeat family, played a vital role in different tumors. Our study found that high‐expression WDR6 promoted survival in lung cancer patients via the Kaplan−Meier plotter and PrognoScan database, the results exhibited that the OS, FP, PPS, and RFS of lung cancer patients were longer in WDR6 high‐expression group than in the low‐expression group, which indicated that WDR6 correlated with a good prognosis for lung cancer patients.

The occurrence and development of the tumor is closely related to the action of immune infiltrating cells in the TEM. The tumor itself is not very immunogenic, but TME inhibits the activity of tumor‐specific T cells that may be present. Recent studies have shown that CD4^+^ T cells can target cancer cells in a variety of ways, either directly by killing cancer cells through cytolysis mechanisms or indirectly by regulating the TME.^11^ In this study, we found that WDR6 expression significantly and positively correlated with the infiltration of CD4^+^ T cells (Suporting Information: Figure S2), further indicating that the upregulation of WDR6 might improve the prognosis survival of lung cancer via regulating the infiltration of CD4^+^ T cells in the TEM. The balance between immunoeffector cells (such as T cells and natural killer cells) and immunosuppressive Treg cells, dendritic cells, myeloid cells, TAM, and monocyte subpopulations in the TEM regulates the immune response to malignant cells. For example, macrophages situated in TME tend to become  TAM to suppress tumor immune response and drive tumor progression, invasion, and metastasis.^12^ In human and mouse colitis‐associated colorectal cancer models, the expression of CCL2, a TAM marker, increases with neoplastic progression.^13^ Targeting and suppression of tumor‐infiltrating macrophages via CCL2/CCR2 signaling was a therapeutic strategy against hepatocellular carcinoma.^14^ Recently, monocytes have emerged as important regulators of cancer development and progression and enabled tumor growth by inhibiting the immune response to cancer.^15^ In our study, the markers of TAMs (CCL2, CD68, and IL10) and monocyte (CD86 and FCGR3A) negatively correlated with the expression level of the WDR6 gene in LUAD patients via using the TIMER and GEPIA2 tools, and in the TISIDB database, the expression of WDR6 significantly and negatively correlated with chemokines (such as CCL2, CCL7, CCL8, and CCL11) in LUAD and LUSC patients. Furthermore, the data analysis of the TISIDB database also verified that WDR6 expression is negatively related to the infiltration of monocyte, thereby suggesting that WDR6 might exert anticancer effects via negatively regulating the infiltration level of monocyte. Therefore, we speculated that the antitumor of the high‐expression level of WDR6 closely correlated with the infiltration of immune cells.

For the first time, our results manifested that the high‐expression level of WDR6 was associated with a good prognosis, and further indicated that WDR6 could serve as a good prognostic biomarker to diagnose and treat lung cancer. Followingly, we further investigated the relationship between WDR6 expression and immune‐infiltrating cells in the TEM, and the results showed that WDR6 expression significantly correlated with immune‐infiltrating cells and their gene markers in lung cancer patients. In addition, we conducted enrichment analyses via integrating the information on WDR6‐binding components and WDR6 expression‐related genes in lung cancer, and results indicated that WDR6 expression might affect the synthesis of ribosomes, the anticancer tumor effects could involve in ribosomes, and its associated pathway (Supporting Information: Figure S3 and S4). Although we still need experiments in vivo and in vitro to confirm our results and explore detailed mechanisms., based on our data analysis, we strongly suggest that researchers in the field of tumor immunology jointly conduct further studies on the role of WDR6 in lung cancer, and gradually clarify the biological function and prognosis of WDR6 in the immune microenvironment of lung patients.

## 5 CONCLUSION

In brief, we concluded that WDR6 is a prognostic molecular biomarker for good survival correlated with immune cell infiltration in lung cancer. This study first offers a relatively comprehensive understanding of the oncogenic roles of WDR6 for lung cancer.

## AUTHOR CONTRIBUTIONS

**Minghe Lv**: Data curation ; formal analysis; investigation; methodology; project administration; software; supervision; validation; visualization; writing—original draft; writing—review and editing.

## CONFLICT OF INTEREST

The author declare no conflict of interest.

## Supporting information

Supplimentary information.

## Data Availability

The datasets generated and analyzed during the current study are available in TCGA, Oncomine, GTEx, GEPIA 2, TIMER, TIMER2.0, PrognoScan, Kaplan−Meier Plotter, TISIDB, and LinkedOmics datasets.
