# Supplementary material for: WD repeat domain 6 as a novelty prognostic biomarker correlates with immune infiltration in lung cancer: A preliminary study
Source: Immun Inflamm Dis. 2022 Aug 17;10(9):e681. doi: 10.1002/iid3.681 (PMC9382870; doi:10.1002/iid3.681)
Supplement: Supplementary file 1 — Supplimentary information. [file IID3-10-e681-s001.docx]

**
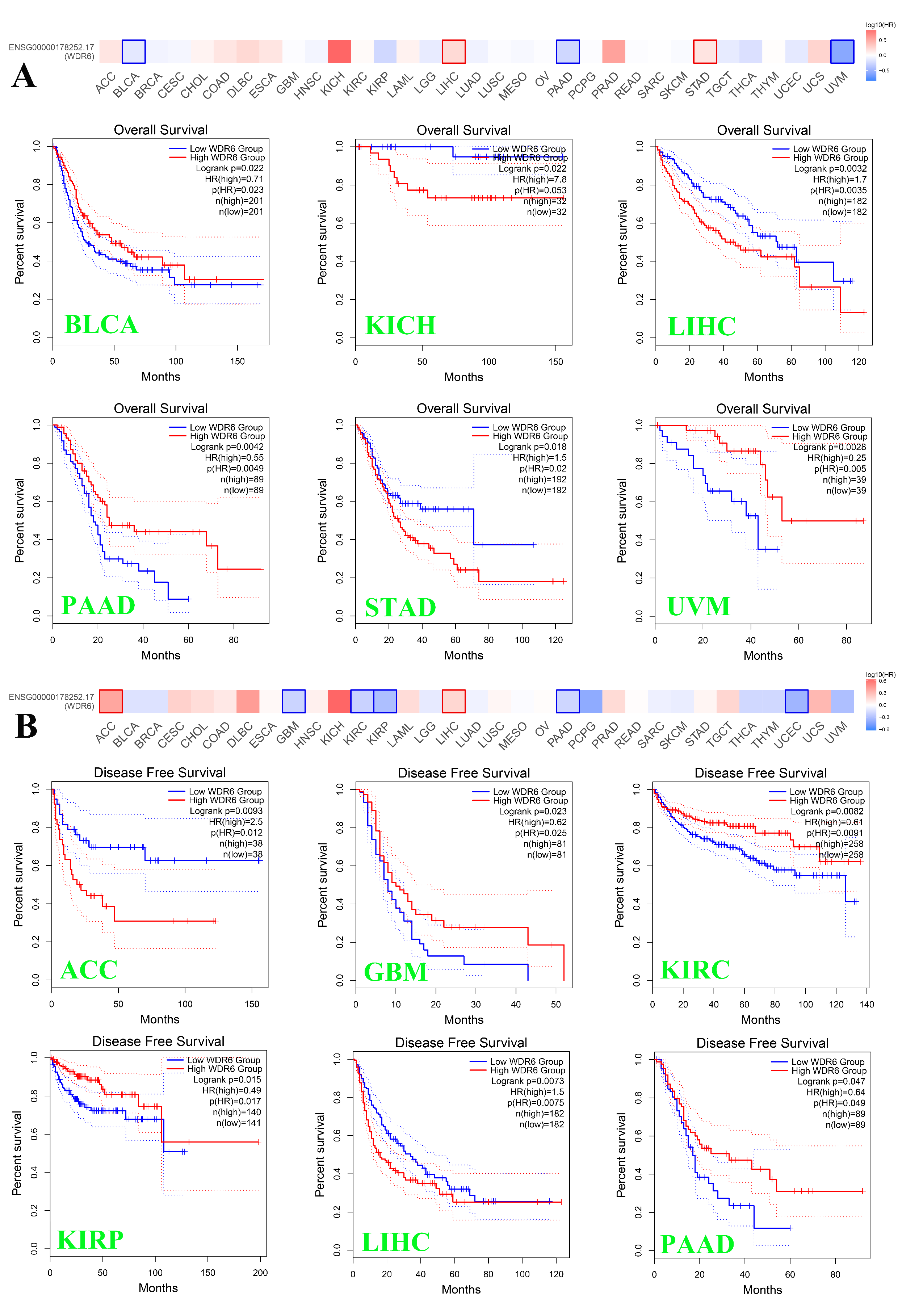
**

**Figure S1 The effects of WDR6 expression on the survival analysis in different tumors.** The heatmap and Kaplan-Meier plot showed the effects of WDR6 expression of pan-cancer on overall survival **(A)** and disease-free survival **(B)** by using the GEPIA2 tool.


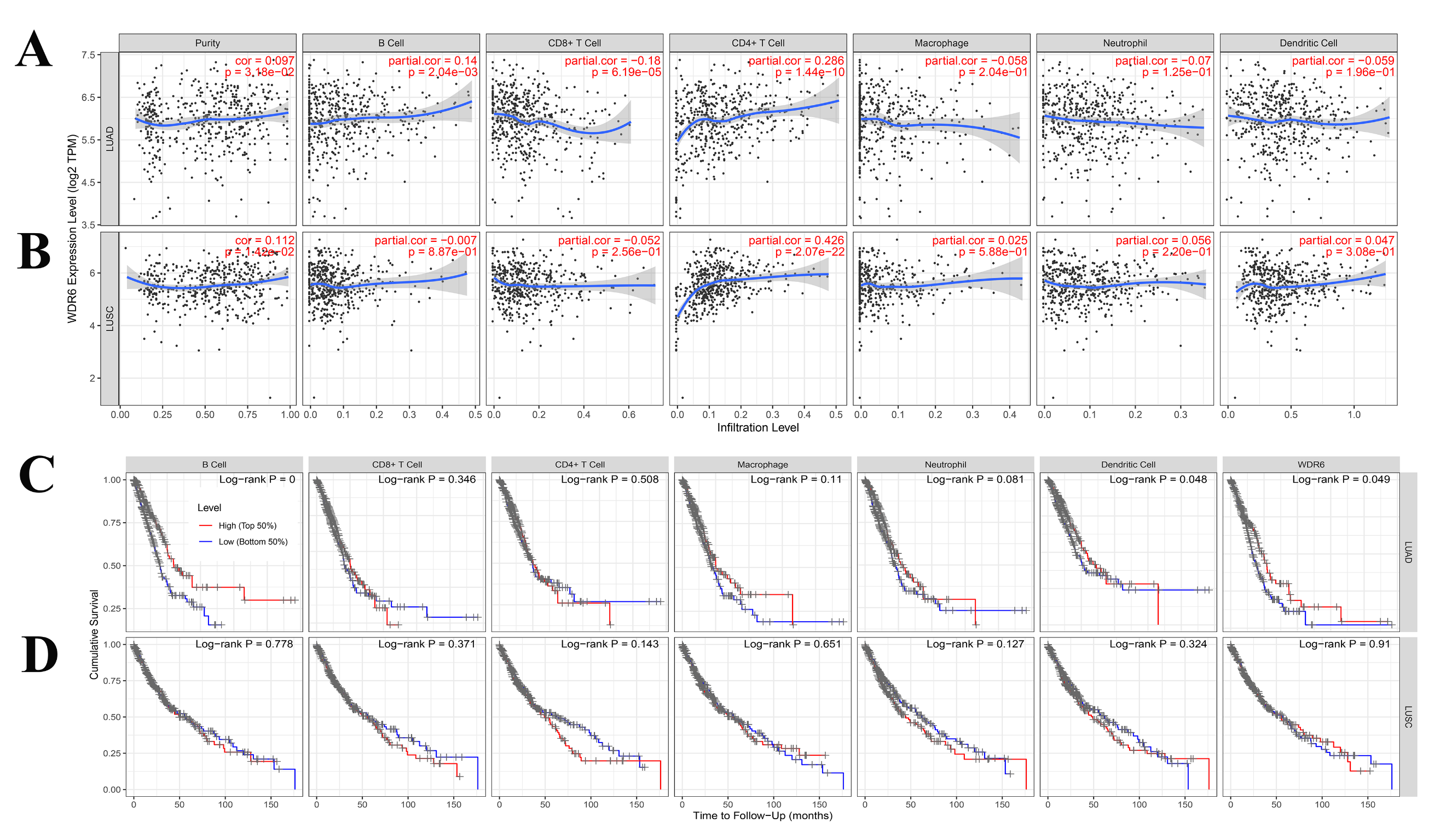


**Figure S2 The correlation and prognosis analysis between WDR6 expression and immune infiltration cells in LUAD and LUSC. (A-B)** Correlation analysis between WDR6 expression and immune infiltration cells in LUAD and LUSC. **(C-D)** Prognosis analysis between WDR6 expression and immne infiltration cells in LUAD and LUSC.

**
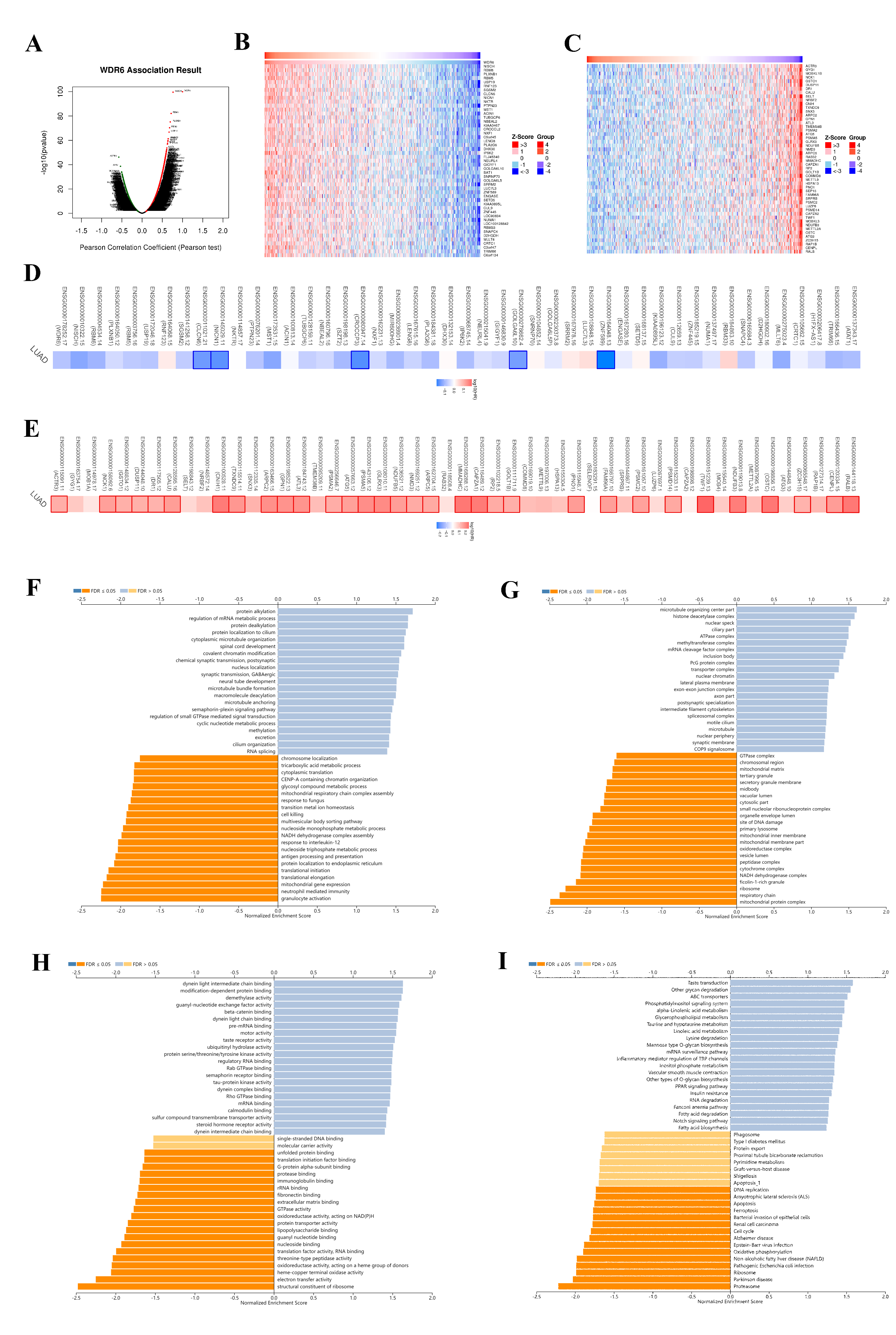
**

**Figure S3 The co-expression genes with WDR6 in LUAD patients from the LinkedOmics database. (A)** The whole significantly associated genes with WDR6 distinguished by Pearson test in LUAD patients. **(B-C)** Top 50 genes positively and negatively correlated with WDR6 in LUAD were respectively displayed by the heatmap. **(D-E)** Survival map of the top 50 genes positively and negatively associated with WDR6 in LUAD. **(F-I)** GO analysis (Biological Process), GO analysis (Molecular Function), GO analysis (Cellular Component), and KEGG pathways of WDR6 in LUAD patients.

**
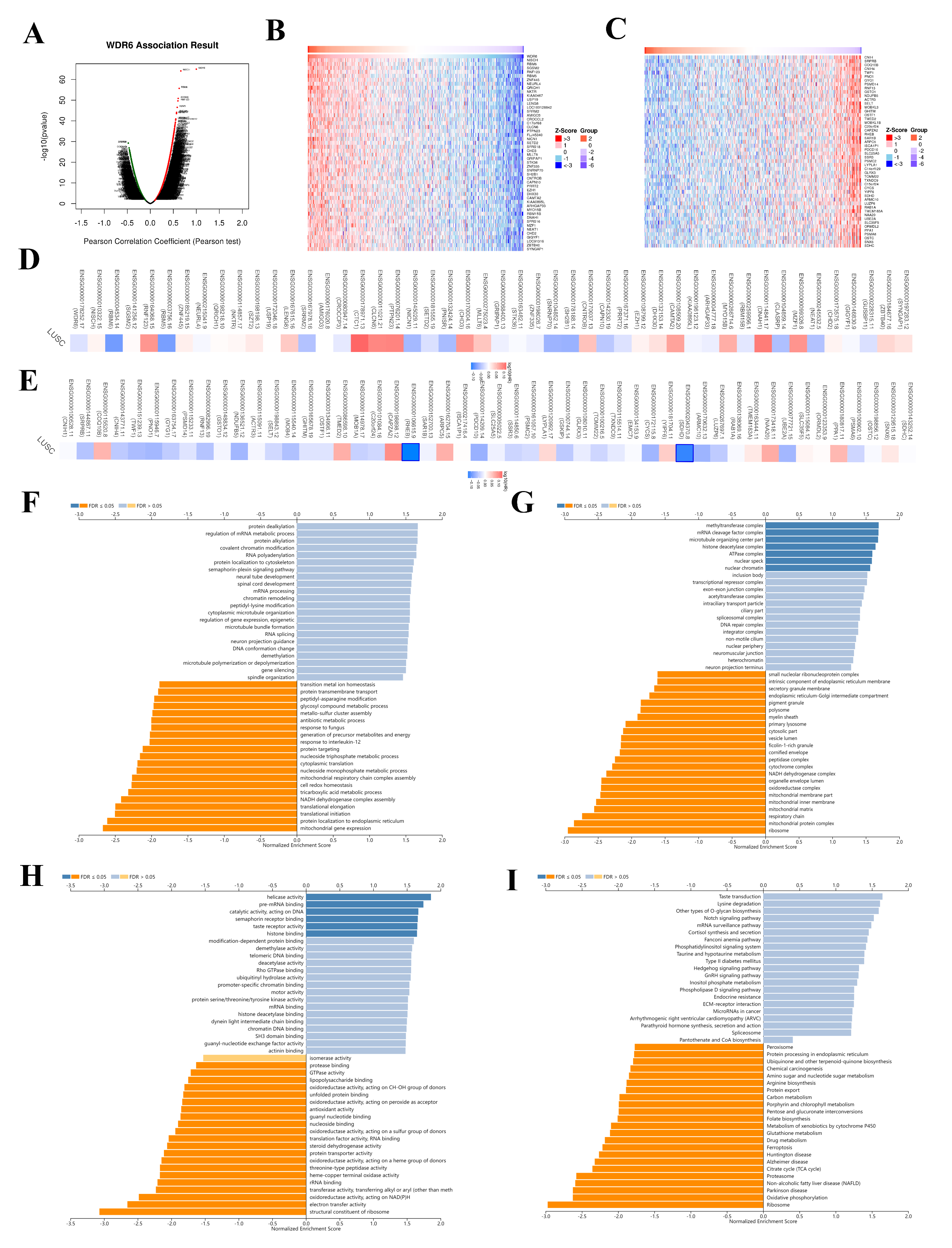
**

**Figure S4 The co-expression genes with WDR6 in LUSC patients from the LinkedOmics database. (A)** The whole significantly associated genes with WDR6 distinguished by Pearson test in LUSC patients. **(B-C)** Top 50 genes positively and negatively correlated with WDR6 in LUSC were respectively displayed by the heatmap. **(D-E)** Survival map of the top 50 genes positively and negatively associated with WDR6 in LUSC. **(F-I)** GO analysis (Biological Process), GO analysis (Molecular Function), GO analysis (Cellular Component), and KEGG pathways of WDR6 in LUSC patients.
